# 2458. Carbapenem-resistant *Acinetobacter baumannii* complex infections and SARS-CoV-2 positivity in 9 U.S. sites, 2020-2022

**DOI:** 10.1093/ofid/ofad500.2076

**Published:** 2023-11-27

**Authors:** Sandra N Bulens, Amanda Hall, Jesse T Jacob, Gillian Smith, Lucy E Wilson, Elisabeth Vaeth, Christopher Wilson, Daniel Muleta, Ghinwa Dumyati, Rebecca Tsay, Meghan Maloney, Nicole Stabach, Ruth Lynfield, Sean O’Malley, Rebecca Pierce, Heather Hertzel, Christopher A Czaja, Helen Johnston, Julian E Grass, Joshua Brandenburg, Alice Guh, Shelley Magill

**Affiliations:** CDC, Atlanta, Georgia; Centers for Disease Control and Prevention/ ORISE, Atlanta, Georgia; Emory University School of Medicine, Atlanta, GA; Georgia Emerging Infections Program, Atlanta, GA; Foundation for Atlanta Veterans Education and Research, Decatur, GA; Atlanta Veterans Affairs Medical Center, Decatur, GA, Atlanta, Georgia; University of Maryland Baltimore County, Baltimore, Maryland; Maryland Department of Health, Baltimore, Maryland, Baltimore, Maryland; Tennessee Department of Health, Nashville, Tennessee; Tennessee Department of Health, Nashville TN, Antioch, Tennessee; New York Emerging Infections Program and University of Rochester Medical Center, Rochester, New York; New York Rochester Emerging Infections Program at the University of Rochester Medical Center, Rochester, NY; Connecticut Department of Public Health, Hartford, Connecticut; Connecticut Department of Public Health, Hartford, CT, Hartford, Connecticut; Minnesota Department of Health, St. Paul, MN; MN EIP, Minneapolis, Minnesota; Oregon Health Authority, Portlant, Oregon; Oregon Public Health Division, Oregon Health Authority; Portland, OR., Portland, Oregon; Colorado Department of Public Health and Environment, Denver, Colorado; Colorado Department of Public Health, Denver, Colorado; CDC, Atlanta, Georgia; Centers for Disease Control and Prevention, Atlanta, GA; CDC, Atlanta, Georgia; CDC, Atlanta, Georgia

## Abstract

**Background:**

Reports have shown increases in carbapenem-resistant *Acinetobacter baumannii* complex (CRAB) infections during the COVID-19 pandemic. To describe the effect of SARS-CoV-2 (SC2) infection on CRAB infection epidemiology, we analyzed data from the Centers for Disease Control and Prevention’s Emerging Infections Program (EIP) population-based CRAB surveillance in 9 U.S. sites.

**Figure:**
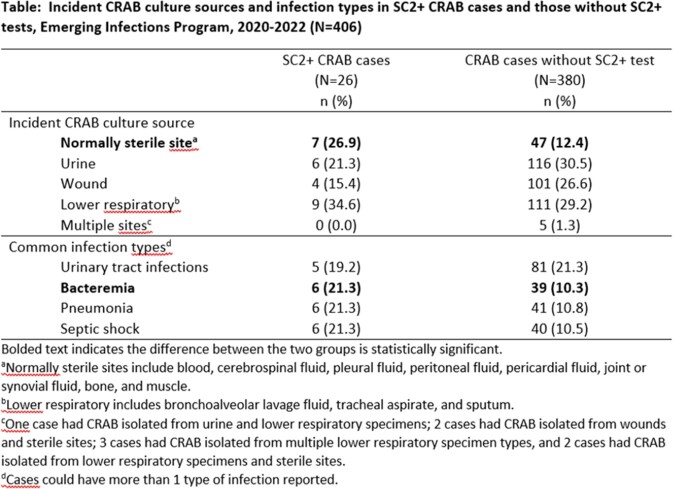

Methods: In 2020–22, among surveillance-area residents, an incident CRAB case was defined as the 1^st^ isolation of *A. baumannii* complex resistant to ≥1 carbapenem (excluding ertapenem) from a normally sterile site or urine (or lower respiratory tract or wound, as of 2021) in a 30-day period. We included cases that underwent chart review. Cases with a culture collected ≥3 days after hospitalization were considered hospital-onset (HO); all others were community-onset (CO). Cases with a SC2+ test ≤14 days before the incident CRAB culture were compared to other CRAB cases.

**Results:**

Of 406 CRAB cases representing 344 patients, 26 (6%) cases in 24 patients were SC2+. SC2+ case-patients were more likely to be female than other CRAB case-patients (58% vs 34%, p=0.02); there were no significant differences in race/ethnicity or age distribution. SC2+ cases were more likely to be obese (42% vs. 23%, p=0.03), have bacteremia (23% vs. 10%, p=0.05), and to have died (39% vs. 16%, p=0.003). SC2+ cases were also more likely to require hospitalization (100% vs. 75%, p=0.005) and have HO CRAB (58% vs 29%, p=0.003). Median hospital length of stay was longer in SC2+ cases (19 days, interquartile range [IQR] 8–30) than in other CRAB cases (14 days, IQR 7–26, p=0.42), but there were no significant differences in the percentages with intensive care unit stays or mechanical ventilation in the 7 days before CRAB culture. Culture sources and infection types in SC2+ cases vs. other CRAB cases are shown in the Table.

**Conclusion:**

A small minority of CRAB patients were infected with SC2+ in the 14 days before CRAB infection but these patients had epidemiological and clinical features that were distinct from other CRAB patients, including higher mortality. Infection control and clinical measures are needed to protect SC2+ patients from secondary bacterial infection and mitigate poor outcomes in those with SC2 and CRAB infection.

**Disclosures:**

**Ghinwa Dumyati, MD**, Pfizer: Grant/Research Support **Rebecca Tsay, MPH**, CDC: Grant/Research Support

